# Expanded Genomic Diversity of Cacao Leafroll Virus Enables Improved Detection Using Multiplex RT-PCR

**DOI:** 10.3390/pathogens15090975

**Published:** 2026-09-14

**Authors:** Ihsan Ullah, Muhammad Kamran, Andrew J. Daymond, Jim M. Dunwell

**Affiliations:** 1 School of Agriculture, Policy and Development, University of Reading, Reading RG6 6EU, UK; i.ullah@reading.ac.uk (I.U.); a.j.daymond@reading.ac.uk (A.J.D.); 2 Sugarcane Research Institute, Ayub Agricultural Research Institute, Faisalabad 38850, Pakistan

**Keywords:** cacao, cacao leafroll virus, *Polerovirus CALRV*, metatranscriptomics, multiplex RT-PCR, virus diagnostics, quarantine

## Abstract

Cacao leafroll virus (CaLRV; species *Polerovirus CALRV*, family *Solemoviridae*), previously referred to as cacao polerovirus, infects cacao (*Theobroma cacao*) germplasm in the Americas. Screening of 287 cacao accessions with three published multiplex RT-PCR assays detected CaLRV in nine accessions but produced reproducibly discordant results for the accession LCT EEN 412. Metatranscriptomic sequencing generated three near-complete CaLRV genome sequences, CaLRV-TSAN792, CaLRV-POUND15A, and CaLRV-LCTEEN412 (5985–5989 nt), from accessions originating from Brazil, Trinidad and Tobago, and Ecuador, respectively. The divergent CaLRV-LCTEEN412 assembly was verified by RT-PCR, cloning, and nanopore sequencing. In addition, analysis of five public cacao RNA-seq datasets yielded one near-complete CaLRV-PA16 assembly included in whole-genome analysis and four fragmented assemblies that informed primer design. Comparison of the four sequences characterised here with six previously reported genomes showed that nine shared 98.8–99.7% pairwise nucleotide identity, whereas CaLRV-LCTEEN412 shared 85.0–85.2%. Primer-binding-site mismatches in CaLRV-LCTEEN412 were consistent with its discordant detection by published assays. Two new multiplex RT-PCR assays were therefore designed from the expanded alignment and evaluated using four CaLRV-positive cacao accessions, including LCT EEN 412. These results expand known CaLRV genomic diversity and provide dual-target tools that reduce dependence on a single locus and enable recognition of target discordance.

## 1. Introduction

Cacao (*Theobroma cacao* L.) is an economically and socially important perennial crop, but the international exchange of cacao germplasm also creates a route for pathogen movement. Safe movement therefore depends on quarantine systems that combine visual observation and biological indexing with pathogen-specific molecular testing. Molecular assays are particularly important when infections are asymptomatic, symptoms are non-specific, or symptom development is delayed. High-throughput sequencing has further expanded the known diversity of cacao-associated viruses and provides the sequence information needed to assess whether established diagnostic assays adequately represent circulating variants [1,2,3].

Cacao leafroll virus (CaLRV; species *Polerovirus CALRV*, family *Solemoviridae*) is a positive-sense, single-stranded RNA virus with a genome of approximately 6 kb. Its compact genome contains overlapping open reading frames (ORFs) and programmed translational events. ORF0 and ORF1 encode P0 and P1, respectively, while a −1 ribosomal frameshift produces the P1-P2 RNA-dependent RNA polymerase (RdRP). The 3′ region encodes P3a, the P3 coat protein (CP), and the overlapping P4 movement protein (MP), while stop-codon readthrough produces the P3-P5 readthrough protein. This organisation permits multiple proteins to be expressed from a small genome, but conservation of protein function does not necessarily ensure conservation of the underlying nucleotide sequences required for primer binding [4,5].

The virus characterised in our earlier study was reported as cacao polerovirus (CaPV) [2]. Subsequent reports used the name cacao leafroll virus [5,6], and the current ICTV classification assigns the exemplar OR423049 to species *Polerovirus CALRV* [7]. CaLRV is therefore used throughout this manuscript. Three multiplex RT-PCR assays were previously developed for detection, each combining one virus-specific target in the RdRP, MP, or readthrough region with a cacao internal control. Used as a panel, these assays sampled multiple viral loci and could reveal discordant detection. However, they were designed from the limited sequence diversity available at the time. Their performance against divergent CaLRV variants was therefore unknown, and dependence on any single virus-specific locus could permit primer-site variation to produce inefficient or false-negative detection.

In this study, 287 cacao accessions were screened using the three published multiplex RT-PCR assays. Reproducibly discordant detection in the Ecuadorian accession LCT EEN 412 prompted metatranscriptomic sequencing and independent sequence verification. Three newly generated CaLRV genomes were analysed with previously reported genomes and sequences recovered from public cacao RNA-seq datasets to characterise genomic and phylogenetic diversity and to evaluate published primer-binding sites. The expanded sequence alignment was then used to develop and evaluate two dual-target multiplex RT-PCR assays intended to reduce dependence on a single viral locus. A defined pruning-tool exposure was also assessed as supporting evidence relevant to quarantine management.

## 2. Materials and Methods

### 2.1. RNA Extraction and cDNA Synthesis

Two hundred and eighty-seven cacao accessions maintained at the International Cocoa Quarantine Centre, Reading, UK (ICQC-R), and listed in Supplementary Table S1 were sampled for CaLRV screening. For each accession, two 8-mm discs were collected from each of three fully expanded leaves and pooled. Samples were ground under liquid nitrogen in 2 mL microcentrifuge tubes containing ceramic beads using a TissueLyser II (QIAGEN, Hilden, Germany). Total RNA was extracted using a modified CTAB method, followed by on-column DNase I digestion and purification with the Spectrum Plant Total RNA Kit (Merck, Gillingham, UK), as described previously [2]. RNA concentration and purity were measured using a NanoDrop 2000 spectrophotometer (Thermo Fisher Scientific, Horsham, UK), and RNA integrity was assessed by electrophoresis on a 1.2% agarose gel.

Routine cDNA used for germplasm screening and evaluation of the new dual-target assays was synthesised from 500 ng of total RNA using SuperScript IV VILO Master Mix with ezDNase Enzyme (Thermo Fisher Scientific), according to the manufacturer’s instructions. The resulting cDNA was diluted 1:4 with nuclease-free water. Gene-specific cDNA prepared for assessment and independent verification of the CaLRV-LCTEEN412 assembly is described separately in Section 2.5. Total RNA and cDNA were stored at −80 °C until use.

### 2.2. Germplasm Screening Using Published Multiplex RT-PCR Assays

All 287 cacao accessions were screened using the three previously reported multiplex RT-PCR assays [2]. Each assay included one CaLRV-specific primer pair and a second primer pair targeting the cacao acyl carrier protein 1 (*ACP1*; LOC18599903) transcript as an internal control (IC) for RNA extraction, reverse transcription, and PCR amplification. Each forward–reverse primer pair was premixed, with each primer at a concentration of 5 µM.

Each 20 µL reaction contained 10 µL of Platinum II Hot Start PCR Master Mix (Thermo Fisher Scientific), 1.5 µL of the CaLRV primer-pair mixture, 0.5 µL of the *ACP1* primer-pair mixture, 5 µL of diluted VILO cDNA, and 3 µL of nuclease-free water. Thermal cycling comprised initial denaturation at 94 °C for 2 min; 35 cycles of 94 °C for 10 s, 60 °C for 10 s, and 68 °C for 20 s; and final extension at 68 °C for 2 min.

Products were resolved on 1.5–1.7% agarose gels, stained with ethidium bromide, and compared with a GeneRuler 100 bp DNA Ladder (Thermo Fisher Scientific). Each run included a CaLRV-positive control, the CaLRV-negative cacao control EET 272 [ECU] (RUQ 6), and a no-template control (NTC). A result was considered interpretable only when the cacao *ACP1* product was detected in the sample. Any initial CaLRV-positive result was verified by repeating the three-assay panel using RNA extracted from a newly collected sample. An accession was recorded as CaLRV-positive when a virus-specific product was detected initially and confirmed in the newly collected sample; discordant detection among the three viral targets was recorded separately.

### 2.3. Metatranscriptomic Sequencing, Sequence Data Processing, and Identification of Viral Contigs

Total RNA extracted from three cacao accessions was submitted to Novogene (Cambridge, UK) for metatranscriptomic sequencing. Eukaryotic and prokaryotic ribosomal RNA (rRNA) was depleted, and the remaining RNA was fragmented to approximately 250–300 bp before reverse transcription into double-stranded cDNA, end repair, A-tailing, adapter ligation, fragment-size selection, and PCR amplification. The libraries were sequenced on a NovaSeq X Plus platform (Illumina, San Diego, CA, USA), generating approximately 12 Gb of paired-end raw sequence data (2 × 150 bp) per sample.

Paired FASTQ files were processed using the Galaxy platform (https://galaxy-main.usegalaxy.org/; accessed on 30 May 2026) [8]. Initial and post-trimming sequence quality was examined using FastQC v0.12.1 [9] and summarised using MultiQC v1.35 [10]. Adapter and low-quality sequences were removed using Trimmomatic v0.39 [11] in paired-end mode with the TruSeq3-PE adapter file and the following settings: LEADING:20, TRAILING:20, SLIDINGWINDOW:4:20, and MINLEN:75. Only surviving paired reads were retained.

Residual rRNA reads were removed using SortMeRNA v4.3.6 [12] with the bundled Rfam 5S and 5.8S and SILVA bacterial, archaeal, and eukaryotic rRNA databases. Paired output was enabled so that both reads of a pair were retained in the non-rRNA output when appropriate.

The non-rRNA reads were aligned to the reference genome of cacao accession Criollo (GCF_000208745.1) using Bowtie2 v2.5.5 [13] in very-sensitive-local mode. Read pairs for which both mates remained unmapped were retained for virus discovery and assembled de novo using Trinity v2.15.1 [14], with a minimum contig length of 300 nt.

Assembled contigs were searched using DIAMOND BLASTX v2.2.4 [15] in sensitive mode against a curated plant-virus-focused database containing 10,810 viral protein records (Supplementary Data S2). Searches used an E-value threshold of 1 × 10^−5^ and a maximum of ten target sequences per query. Candidate viral contigs were prioritised when they produced alignments of at least 100 amino acids, at least 30% amino acid identity, and consistent matches to one or more proteins from the same virus group.

Quality-filtered reads were mapped back to candidate CaLRV contigs using Bowtie2. Mapped-read counts were obtained from the resulting alignment files, and nucleotide depth was calculated using SAMtools depth v1.22 [16]. Coverage was examined across each assembly to identify unsupported regions or localised anomalies, and mean depth was calculated across its full length. The final assemblies recovered from cacao accessions TSAN 792, POUND 15/A, and LCT EEN 412 were designated CaLRV-TSAN792, CaLRV-POUND15A, and CaLRV-LCTEEN412, respectively.

### 2.4. Analysis of Public Cacao RNA-Seq Datasets

Five cacao RNA-seq datasets from BioProject PRJNA558793 [17] were reanalysed. BioSample and SRA run accessions and dataset sizes are provided in Supplementary Table S2. Raw reads were downloaded from the NCBI Sequence Read Archive and processed using the host-read subtraction, de novo assembly, and DIAMOND BLASTX workflow described in Section 2.3. Near-complete assemblies were eligible for whole-genome analyses, whereas fragmented assemblies were used only for primer-site assessment and assay design. Unassembled intervals between non-overlapping contigs were recorded as missing data rather than biological deletions.

### 2.5. Terminal Assessment and Independent Verification of the CaLRV-LCTEEN412 Assembly

Seven overlapping primer pairs were designed from the CaLRV-LCTEEN412 assembly to assess the near-terminal regions: three pairs covering positions 38–306 near the 5′ end and four pairs covering positions 5665–5983 near the 3′ end. The primer pairs were evaluated by RT-PCR using routine VILO cDNA and two gene-specific cDNAs synthesised using L412_5926R5 or L412_5983R6 as reverse-transcription primers. The L412_5926R5-derived cDNA was selected for near-complete-genome amplification, whereas the L412_5983R6-derived cDNA was used to assess the most distal 3′-terminal primer pair. The previously reported movement-protein/*ACP1* multiplex assay and NTCs were included as controls. Primer sequences, detailed reaction conditions, and the terminal-assessment gel are provided in Supplementary Method S1, Supplementary Table S12, and Supplementary Figure S1, respectively.

For independent sequence verification, cDNA synthesised using L412_5926R5 as the reverse-transcription primer was amplified with the L412_38F1/L412_5926R5 primer pair, which was designed to amplify a 5889-nt region spanning positions 38–5926 of the CaLRV-LCTEEN412 assembly. The amplicon was purified and cloned, and recombinant plasmid DNA was subjected to nanopore long-read sequencing by Source BioScience (Nottingham, UK). The resulting insert consensus sequence was aligned with the metatranscriptomic assembly to determine sequence coverage and nucleotide identity. Detailed cDNA synthesis, amplification, cloning, and sequencing procedures are provided in Supplementary Method S1.

### 2.6. Genome Annotation and Comparative Analyses

The three newly generated CaLRV genomes and the near-complete CaLRV-PA16 assembly recovered from public RNA-seq data were annotated using OR423049 as the reference. ORFs and their predicted products were identified using NCBI ORFfinder (https://www.ncbi.nlm.nih.gov/orffinder/; accessed on 4 June 2026). The ORF1-ORF2 and ORF3-ORF5 products were translated by applying the established −1 ribosomal frameshift and stop-codon readthrough mechanisms, respectively. Start and stop codons, the frameshift site, the ORF3 readthrough stop codon, and all predicted proteins were verified manually.

Whole-genome analyses included ten complete or near-complete CaLRV sequences aligned using MUSCLE [18] in MEGA 12.1 [19,20]. Pairwise whole-genome nucleotide identities were calculated using Sequence Demarcation Tool version 1.2 [21]. The reference genome also provided the common coordinate system for reference-based comparisons. For the four sequences characterised here, nucleotide substitutions and indels were identified from the whole-genome alignment. Each retained contiguous gap was counted as one indel event, and its length was recorded separately. Terminal gaps attributable to incomplete UTR sequences were treated as missing data and excluded, whereas coding-region differences were retained because the coding sequences were complete.

Feature-level comparisons were conducted separately for the two UTRs, the intergenic region, the individual ORFs, and the programmed fusion products. Nucleotide and amino acid identities were calculated across assessable aligned positions, with one-sided internal gaps scored as differences.

For codon-aware comparisons, nucleotide differences within gap-free aligned codon pairs were classified according to whether the two codons encoded the same or different amino acids. Gap-containing codons were recorded separately. Each annotated product was analysed independently, and counts from overlapping reading frames were not combined.

### 2.7. Recombination Screening and Phylogenetic Analysis

The 6048-position nucleotide alignment of ten complete or near-complete CaLRV genomes was screened for evidence of recombination using GARD v0.2 [22] through the Datamonkey web server (https://www.datamonkey.org/gard; accessed on 30 July 2026) [23], in standard mode with default settings. To assess the influence of the divergent CaLRV-LCTEEN412 genome, the analysis was repeated after excluding this sequence while retaining the same alignment-coordinate system. Competing breakpoint models were compared using the corrected Akaike information criterion (c-AIC). Inferred breakpoint positions were mapped against the annotated genomic features to determine whether any occurred within the ORF3 coat protein coding region. Permanent links to both analyses are provided in Supplementary Table S7.

Maximum-likelihood coat protein phylogenies were reconstructed in MEGA 12.1 [20]. Substitution models were evaluated separately for the amino acid and nucleotide alignments, and the model with the lowest Bayesian information criterion (BIC) score was selected for each. For each analysis, the initial tree for the heuristic search was selected by comparing Neighbour-Joining and Maximum Parsimony starting trees and retaining the tree with the higher log likelihood.

The amino acid dataset comprised 15 CaLRV sequences, 10 representative sequences from related viruses, and pea enation mosaic virus 1 (PEMV1; NC_003629) as the outgroup. The original alignment contained 212 positions. The tree was inferred using the JTT+G+I model with five discrete gamma categories, an estimated gamma shape parameter of 1.9895, and an invariant-site proportion of 0.0837. Partial deletion with a 90% site-coverage cutoff retained 196 positions for analysis. Branch support was assessed using 1000 bootstrap replicates.

The nucleotide dataset comprised ORF3 sequences from 15 CaLRV isolates and contained 606 aligned nucleotide positions. The tree was inferred using the Kimura 2-parameter model. Partial deletion with a 90% site-coverage cutoff retained 603 positions for analysis, and branch support was assessed using 1000 bootstrap replicates. No external outgroup was included, and the tree was treated as unrooted.

### 2.8. Primer-Site Analysis and Development of Dual-Target Multiplex RT-PCR Assays

Binding sites of published CaLRV-specific diagnostic primers, broader polerovirus primers, and primers used for CaLRV genome characterisation [2,6,7,24] were mapped against the expanded 14-sequence alignment. IUPAC-degenerate bases compatible with the aligned nucleotide were scored as matches, whereas primer positions falling within unassembled regions of the fragmented public-data assemblies were recorded as not assessable. New primer pairs were designed using Primer3Plus (https://www.primer3plus.com/index.html; accessed on 14 July 2026) [25] from regions conserved across the assessable sequences. CaLRV-specific primer coordinates and predicted amplicon sizes were determined relative to OR423049.

The previously reported MP primer pair [2] was retained and combined with newly designed primer pairs targeting the RdRP and CP/readthrough regions and with the cacao *ACP1* IC primer pair. Candidate CaLRV primer pairs were first evaluated individually using routine VILO cDNA prepared from LCT EEN 412 and then assessed in five multiplex combinations. Combinations producing clearly resolvable expected products without additional amplicons were selected. The development-stage combinations are detailed in Supplementary Table S9. Candidate and retained primer sequences, using their original development-set identifiers, are listed in Supplementary Table S11.

Assay A, designated the primary screening configuration, combined the RdRP and MP targets with the *ACP1* IC. Assay B, designated the complementary configuration, combined the RdRP and CP/readthrough targets with the same IC. Primer sequences, coordinates, target regions, and predicted product sizes for the selected assays are provided in Table 1.

The selected assays used the PCR master mix, thermal-cycling programme, and agarose-gel electrophoresis conditions described in Section 2.2. Each 20 µL reaction contained 10 µL of Platinum II Hot Start PCR Master Mix, 1.5 µL of each of the two CaLRV primer-pair mixtures, 0.5 µL of the *ACP1* primer-pair mixture, 5 µL of 1:4-diluted VILO cDNA, and 1.5 µL of nuclease-free water.

Both assays were evaluated using four CaLRV-positive cacao accessions: LCT EEN 412, TSH 1188, POUND 15/A, and TSAN 792. Accession EET 272 [ECU] served as the CaLRV-negative cacao control, and an NTC was included in each run.

### 2.9. Assessment of Potential Mechanical Transfer During Pruning

Eight three-month-old seedlings of the CaLRV-susceptible cacao clone Amelonado were used to assess potential transfer during pruning. The seedlings did not have RUQ identifiers because they were maintained solely for internal seed production and were not accessioned for distribution. Immediately before pruning-tool exposure, the eight recipient seedlings and the TSH 1188 source plant were tested using all three published multiplex RT-PCR assays described in Section 2.2 to confirm their CaLRV-negative and CaLRV-positive status, respectively. Secateurs were used first to prune TSH 1188 and then immediately, without intervening disinfection, to prune the eight Amelonado seedlings one after another.

The exposed seedlings were maintained in a quarantine tunnel for 24 months under routine monitoring for known or presumptive CaLRV vectors. Samples were collected at three-month intervals from 3 to 24 months after pruning. Samples were processed as described in Section 2.1 and tested using all three published multiplex RT-PCR assays described in Section 2.2.

## 3. Results

### 3.1. Screening of Cacao Germplasm Reveals Discordant Detection in an Ecuadorian Accession

Screening with three previously reported multiplex RT-PCR assays [2] detected CaLRV in nine of 287 cacao accessions representing 13 germplasm sources from 12 countries. Three of 273 post-quarantine accessions, one of nine quarantine accessions, and all five pre-quarantine accessions were tested positive (Table 2). The complete screening dataset is provided in Supplementary Table S1.

**Figure 1:**
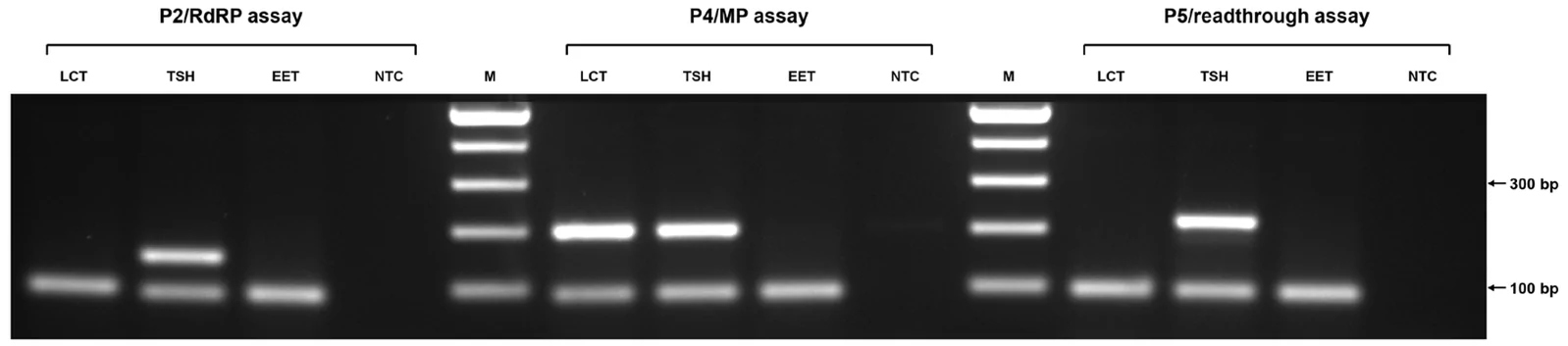
Representative multiplex RT-PCR profiles showing discordant detection of cacao leafroll virus (CaLRV) in LCT EEN 412 using three published assays. The virus-specific amplicons represented P2/RdRP (150 bp), P4/MP (201 bp), and P5/readthrough (212 bp); the cacao *ACP1* internal-control amplicon (IC) was 90 bp. LCT, TSH, and EET denote cacao accessions LCT EEN 412, TSH 1188, and EET 272 [ECU], respectively; TSH and EET served as the CaLRV-positive and CaLRV-negative controls, respectively. NTC, no-template control; M, GeneRuler 100 bp DNA Ladder. RdRP, RNA-dependent RNA polymerase; MP, movement protein; P5, readthrough domain; *ACP1*, acyl carrier protein 1.

Ecuadorian accession LCT EEN 412 produced reproducibly discordant results. The MP-targeting assay generated the expected CaLRV-specific amplicon, whereas the other two yielded only the IC amplicon (Figure 1).

### 3.2. Near-Complete CaLRV Genomes Recovered from Three Cacao Accessions

Metatranscriptomic sequencing and de novo assembly of reads generated for cacao accessions TSAN 792, POUND 15/A, and LCT EEN 412 recovered one near-complete CaLRV genome from each accession. The assemblies were designated CaLRV-TSAN792, CaLRV-POUND15A, and CaLRV-LCTEEN412, respectively. Read mapping provided coverage at every nucleotide position in each assembly. CaLRV-TSAN792 was 5989 nt and was supported by 4655 mapped reads at a mean depth of 117×; CaLRV-POUND15A was 5985 nt, with 596 reads at 15×; and CaLRV-LCTEEN412 was 5988 nt, with 36,125 reads at 905×. The corresponding sequences were deposited in GenBank as PZ760203, PZ760204, and PZ760205, respectively, under BioProject PRJNA1503048.

**Figure 2:**
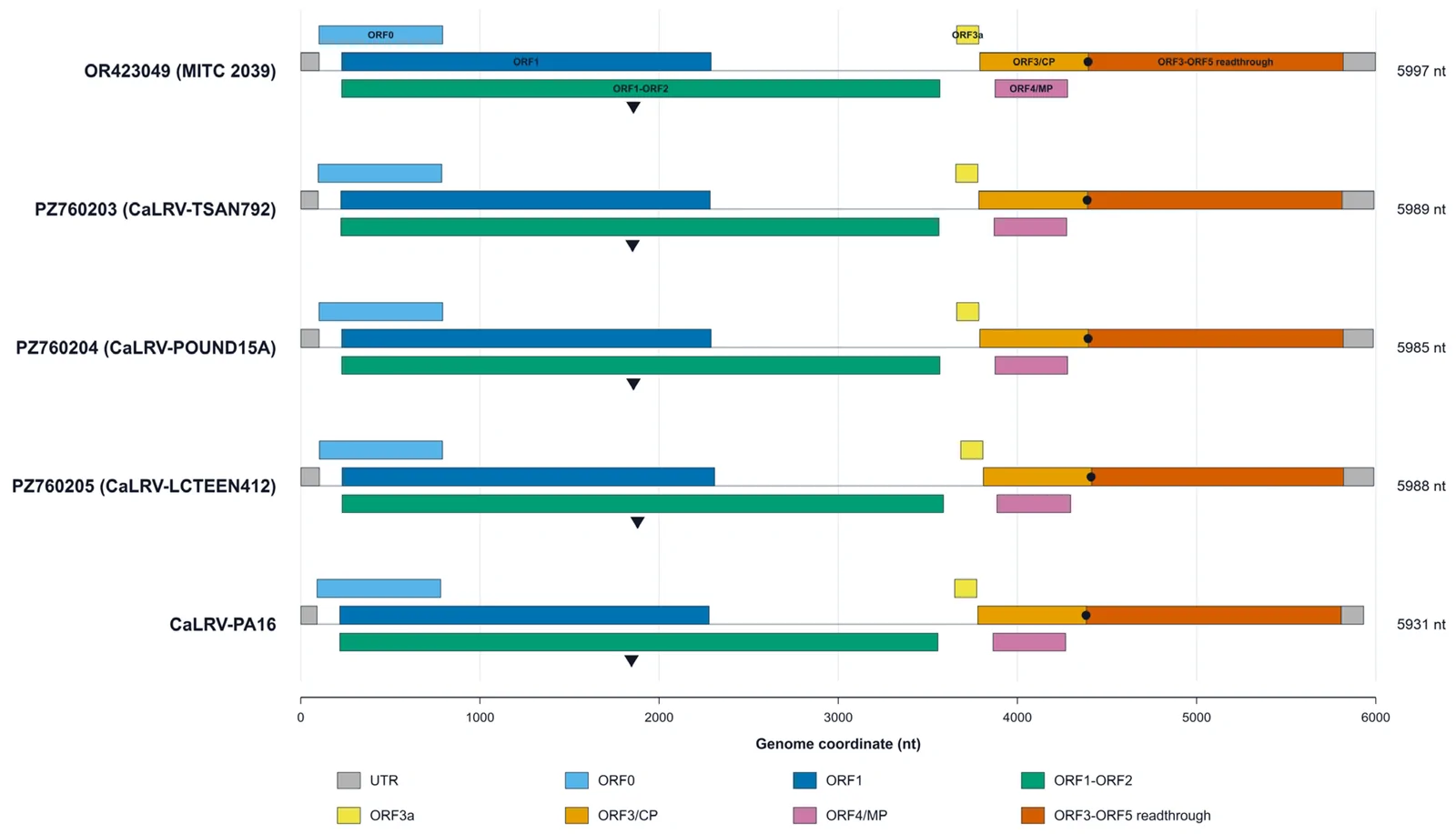
Genome organisation of complete and near-complete cacao leafroll virus (CaLRV) genomes. Genomic features are drawn to scale according to their annotated coordinates. GenBank accession numbers are followed in parentheses by their corresponding isolate designations; CaLRV-PA16 denotes the data-mined assembly recovered from the PA 16 dataset. Black triangles indicate the programmed −1 ribosomal frameshift sites, and black circles indicate the ORF3 readthrough stop codons. CaLRV, cacao leafroll virus; ORF, open reading frame; UTR, untranslated region; CP, coat protein; MP, movement protein.

All three genomes exhibited the expected polerovirus organisation and contained intact ORF0, ORF1, ORF2, ORF3a, ORF3, ORF4, and ORF5 (Figure 2 and Table 3). The programmed −1 ribosomal frameshift site required for expression of the ORF1-ORF2 fusion protein and the ORF3 readthrough stop codon associated with expression of the ORF3-ORF5 readthrough protein were conserved in all three genomes. No premature stop codons were identified.

### 3.3. Independent Verification of the CaLRV-LCTEEN412 Assembly

The near-terminal regions of the CaLRV-LCTEEN412 assembly were first assessed by RT-PCR using seven primer pairs and three cDNA preparations. Six primer pairs generated clear products of the expected sizes. The most distal pair, located entirely within the 3′ UTR at positions 5839–5983, produced only a faint product from VILO cDNA and no detectable product from either gene-specific cDNA preparation (Supplementary Figure S1).

Sequence-based verification was then undertaken as a separate long-amplicon experiment designed to assess most of the assembled genome rather than determine its extreme termini. The cDNA synthesised using L412_5926R5 was amplified with the L412_38F1/L412_5926R5 primer pair, generating the expected 5889-nt product spanning positions 38–5926. This region excluded the first 37 nt of the assembled 5′ end and the final 62 nt of the 3′ end. Nanopore sequencing of the cloned amplicon generated a consensus covering 98% of the metatranscriptomic assembly and sharing 99.37% nucleotide identity with it. The long-read consensus therefore independently verified the assembly across the amplified near-complete genomic region but did not independently determine the extreme terminal sequences. The nanopore insert consensus is provided in Supplementary Data S3.

### 3.4. Public Cacao RNA-Seq Datasets Yield Additional CaLRV Sequences

CaLRV sequences were recovered from all five public cacao RNA-seq datasets. The PA 16 dataset yielded a 5931-nt near-complete assembly, designated CaLRV-PA16, which was included in whole-genome analyses. Among the fragmented assemblies, the NA 70 and NA 34 datasets each yielded two non-overlapping contigs, with combined lengths of 5817 and 5449 nt and reference-coordinate spans of 13–5942 and 25–5590, respectively. The GU 123/V and SPEC 54/1 datasets each yielded three non-overlapping contigs, with combined lengths of 5816 and 4979 nt and reference-coordinate spans of 12–5897 and 49–5897, respectively (Supplementary Table S2 and Supplementary Data S1).

Sequence information from all five datasets contributed to the expanded alignment used for primer-site assessment and assay design, whereas the four fragmented assemblies were excluded from whole-genome identity and variant-count analyses.

### 3.5. Comparative Analyses Identify CaLRV-LCTEEN412 as a Divergent Variant

**Figure 3:**
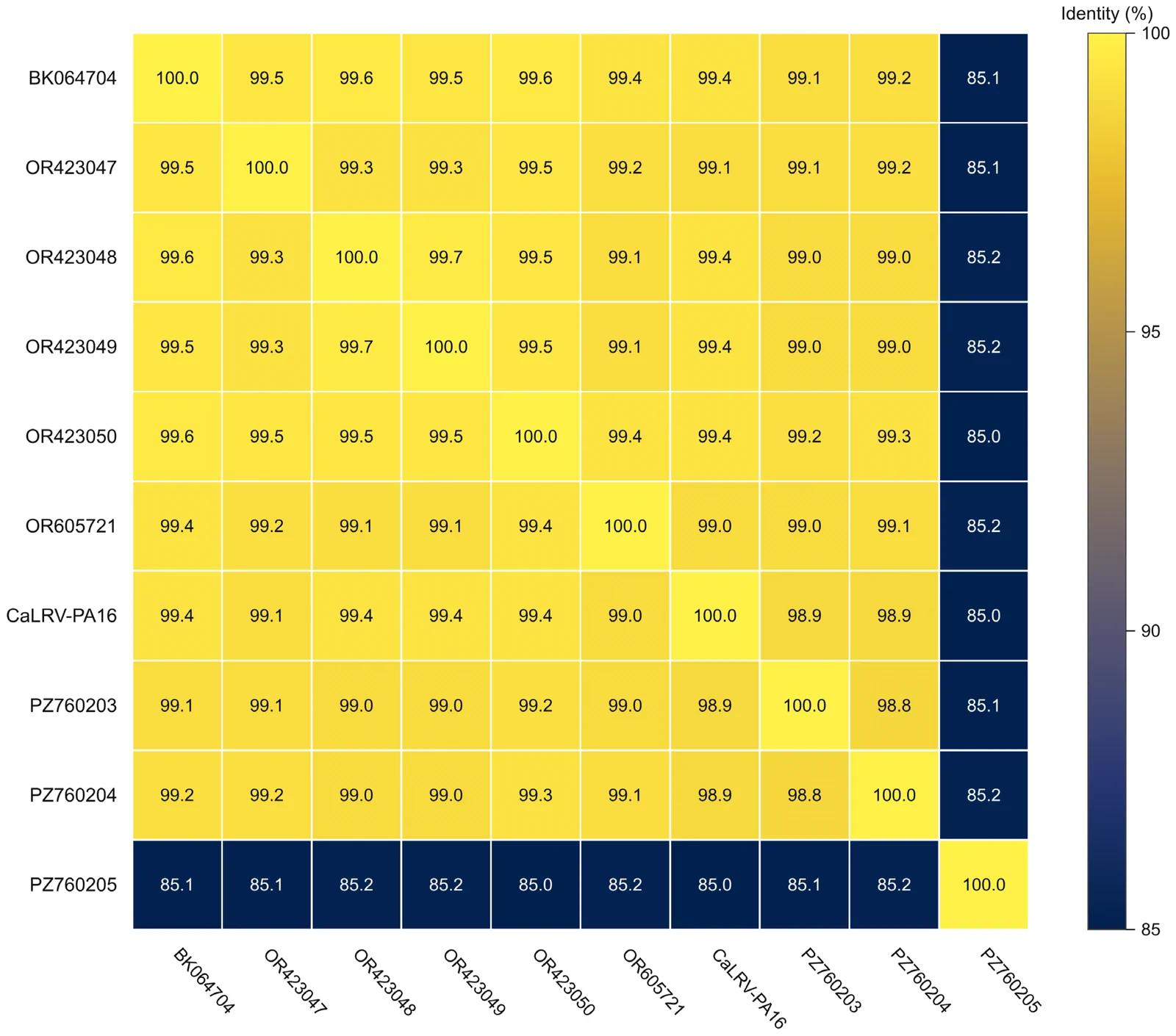
Pairwise whole-genome nucleotide identities among ten complete or near-complete cacao leafroll virus (CaLRV) genomes, calculated from the 6048-position nucleotide alignment using the Sequence Demarcation Tool. Values within cells are percentages. CaLRV-PA16 denotes the near-complete assembly recovered from the public PA 16 dataset. GenBank accessions PZ760203, PZ760204, and PZ760205 represent CaLRV-TSAN792, CaLRV-POUND15A, and CaLRV-LCTEEN412, respectively, generated in this study.

Among the ten complete or near-complete CaLRV genomes, nine formed a closely related group sharing 98.8–99.7% pairwise nucleotide identity. CaLRV-LCTEEN412 shared only 85.0–85.2% identity with these genomes (Figure 3 and Supplementary Table S3).

Relative to OR423049, CaLRV-TSAN792, CaLRV-POUND15A, and CaLRV-PA16 contained 61, 65, and 33 nucleotide substitutions, respectively, and no internal indels. CaLRV-LCTEEN412 contained 911 substitutions and 23 internal indel events affecting 98 nt, comprising 11 insertion events affecting 51 nt and 12 deletion events affecting 47 nt (Supplementary Table S4).

Feature-level comparisons showed that divergence in CaLRV-LCTEEN412 was distributed unevenly across the genome (Table 3; Supplementary Tables S5 and S6). Nucleotide identity ranged from 52.43% in the 5′ UTR to 94.40% in ORF4, whereas amino acid identity ranged from 77.70% for P1 to 93.07% for P3.

Codon-aware comparisons identified both synonymous and amino-acid-changing variation in CaLRV-LCTEEN412, including within the overlapping ORF3 and ORF4 products. Counts for each annotated product are provided in Supplementary Table S6. Because each product was assessed independently, counts from overlapping ORFs are not additive.

### 3.6. Recombination Screening and Coat Protein Phylogenies

In the ten-genome analysis, GARD favoured a multiple-tree model containing nine putative breakpoints over the single-tree multiple-partition model (Delta c-AIC = 467.875). Excluding CaLRV-LCTEEN412 reduced the number of candidate breakpoint positions considered from 978 to 142 and again yielded nine putative breakpoints (Delta c-AIC = 413.889), although their inferred positions shifted. Neither analysis placed a breakpoint within the ORF3 interval at alignment positions 3835–4440; therefore, the coat protein region was analysed as a single phylogenetic partition (Supplementary Table S7).

**Figure 4:**
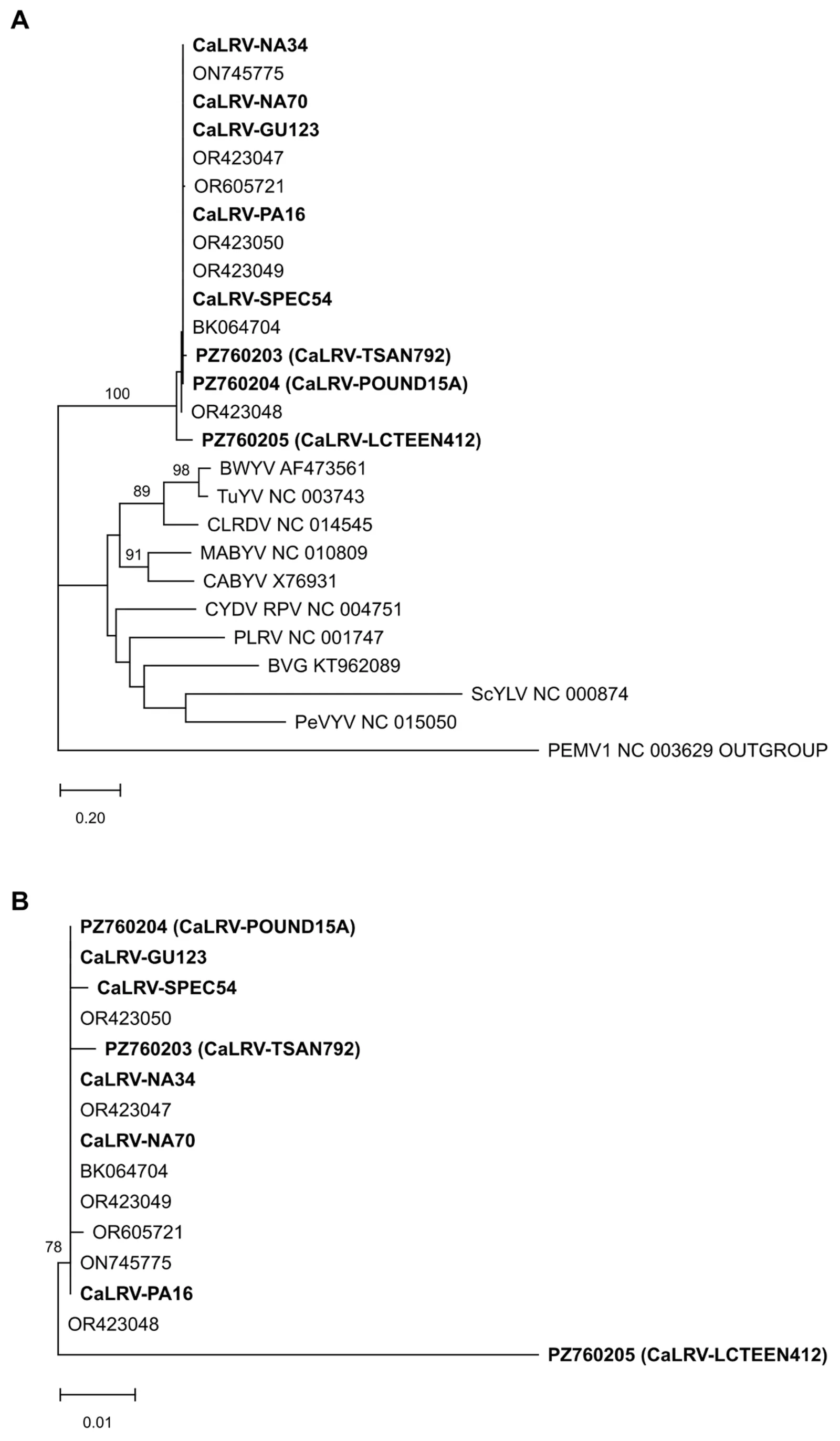
Maximum-likelihood phylogenies based on cacao leafroll virus (CaLRV) coat protein sequences. (**A**) Amino acid tree inferred from 26 sequences, comprising 15 CaLRV sequences, 10 representative sequences from related viruses, and pea enation mosaic virus 1 (PEMV1; NC_003629) as the outgroup. The analysis used 196 amino acid positions retained after partial deletion, the JTT+G+I model with five discrete gamma categories, and 1000 bootstrap replicates. (**B**) Unrooted nucleotide tree inferred from 15 CaLRV sequences and 603 nucleotide positions retained after partial deletion using the Kimura 2-parameter model and 1000 bootstrap replicates. Bootstrap values of 75% or greater are shown for resolved internal branches and rounded to the nearest whole percentage. Branch lengths indicate substitutions per site. Sequences generated or assembled in this study are shown in bold. GenBank accession numbers for genomes generated in this study are followed in parentheses by their isolate designations; labels beginning with CaLRV- and lacking an accession denote assemblies recovered from public RNA-seq datasets.

In the amino acid tree, all 15 CaLRV sequences formed a strongly supported clade (100% bootstrap support) distinct from the representative related viruses (Figure 4A). CaLRV-LCTEEN412 occupied a distinct, longer branch within this clade, whereas the subgroup containing the remaining 14 CaLRV sequences received 67.8% bootstrap support. Identical or nearly identical P3 sequences produced zero-length or very short branches, leaving most relationships within this subgroup unresolved.

The nucleotide tree similarly separated CaLRV-LCTEEN412 from the other CaLRV sequences by a markedly longer branch (Figure 4B). Among the 13 other sequences covering the complete ORF3 region, its sequence differed at 40–43 aligned nucleotide positions and shared 92.9–93.4% pairwise nucleotide identity with them. Ten complete ORF3 sequences were identical. A subgroup of 13 closely related sequences received 78% bootstrap support, but the scarcity of informative sites prevented reliable resolution of most relationships within this subgroup.

### 3.7. Expanded Sequence Diversity Informs Dual-Target Multiplex RT-PCR Assay Design

Published primer-binding sites were assessed across the 14-sequence alignment to place the new assay design in the context of available CaLRV and broader polerovirus primers (Supplementary Table S8). Among the three previously reported CaLRV-specific diagnostic pairs [2], the MP pair P4 was conserved across all 14 sequences. In LCT EEN 412, the P2 and P5 pairs contained four and eight mismatches, respectively, including a mismatch within the final three nucleotides of the P5 forward primer. This variation was consistent with the discordant detection pattern, although the effect of individual mismatches on primer performance was not tested separately.

The broad polerovirus primers PoconF and PococpR, originally reported and subsequently used for CaLRV detection [6,24], contained two or three non-degenerate mismatches across the CaLRV sequences, none within the final three primer nucleotides. Among primers reported for CaLRV genome characterisation [7], CaPol_1335R contained two 5-prime-proximal mismatches in LCT EEN 412, whereas CaPol_653R contained a mismatch at the penultimate 3-prime nucleotide. CaPol_5225F contained multiple mismatches, including substitutions at its terminal and penultimate 3-prime nucleotides. These terminal changes could impair amplification or terminal-sequence recovery from LCT EEN 412, but this predicted effect was not tested experimentally.

The expanded alignment was used directly to select the new primer sites; conservation across the 14 sequences was therefore a design criterion rather than a retrospective finding. The common RdRP target provided a conserved upstream locus, whereas the alternative MP and CP/readthrough targets provided independent downstream detection sites.

### 3.8. Selection and Evaluation of Two Multiplex RT-PCR Assays

All candidate CaLRV primer pairs generated products of the expected sizes when tested individually using LCT EEN 412 cDNA. Five multiplex combinations were then evaluated (Supplementary Table S9). The three combinations containing both downstream viral primer pairs produced an additional band consistent with unintended cross-pair amplification and were excluded.

**Figure 5:**
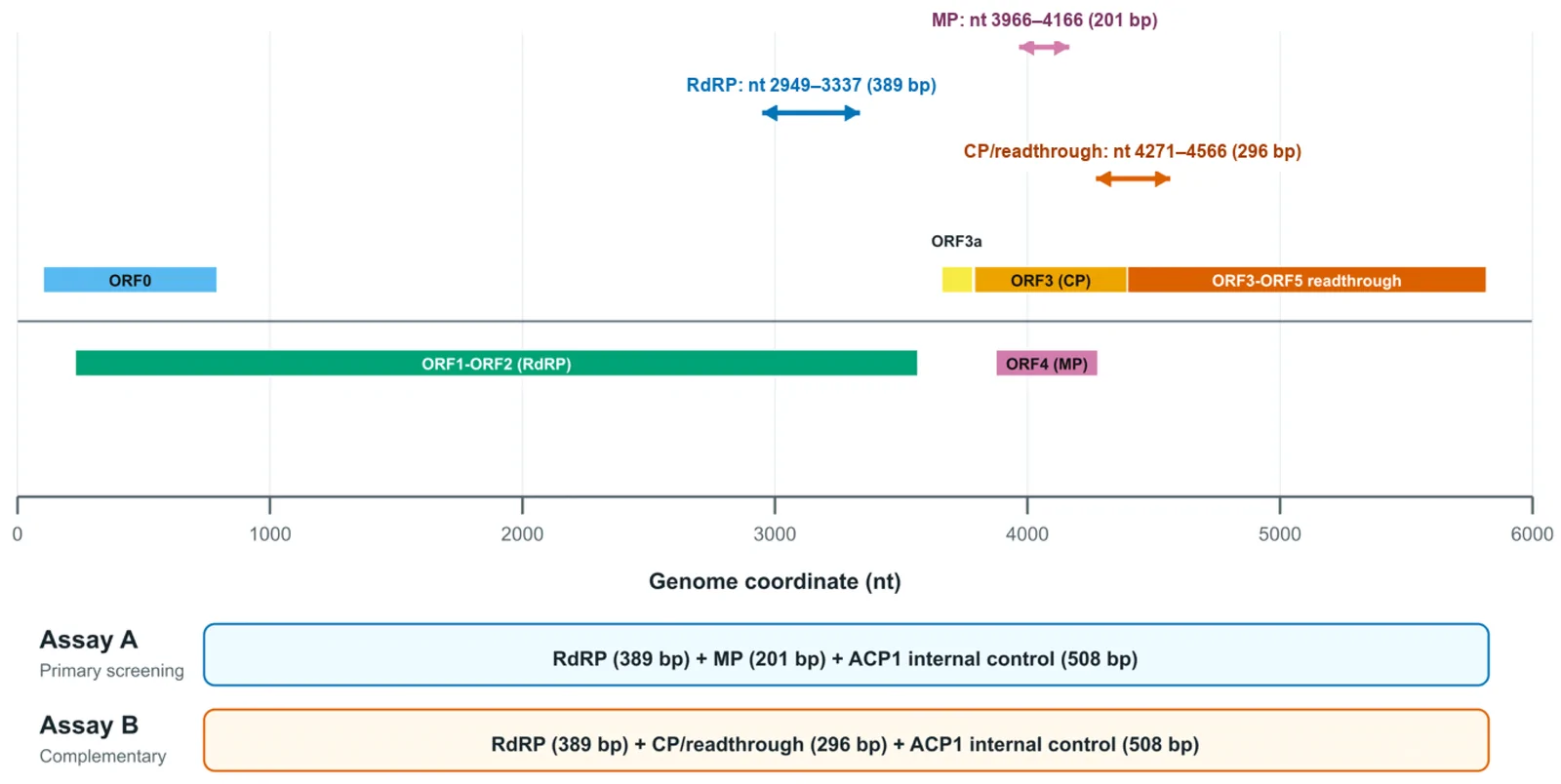
Genomic positions and target composition of the selected dual-target multiplex RT-PCR assays designed for detection of cacao leafroll virus. Assay A, the primary screening configuration, combines targets in the RNA-dependent RNA polymerase (RdRP; nt 2949–3337, 389 bp) and movement protein (MP; nt 3966–4166, 201 bp) regions with the cacao acyl carrier protein 1 (*ACP1*; 508 bp) internal control. Assay B, the complementary configuration, combines the same RdRP and *ACP1* targets with a coat protein/readthrough-region target (CP/readthrough; nt 4271–4566, 296 bp). Viral coordinates and predicted amplicon sizes are relative to OR423049. *ACP1* is a cacao target and is therefore not mapped on the viral genome. bp, base pairs; nt, nucleotides; ORF, open reading frame.

Two combinations generated clear, readily distinguishable products without additional bands and were retained as assay A (development combination 4) and assay B (development combination 5). Their target composition and genomic positions are shown in Figure 5, and the retained primer details are listed in Table 1.

**Figure 6:**
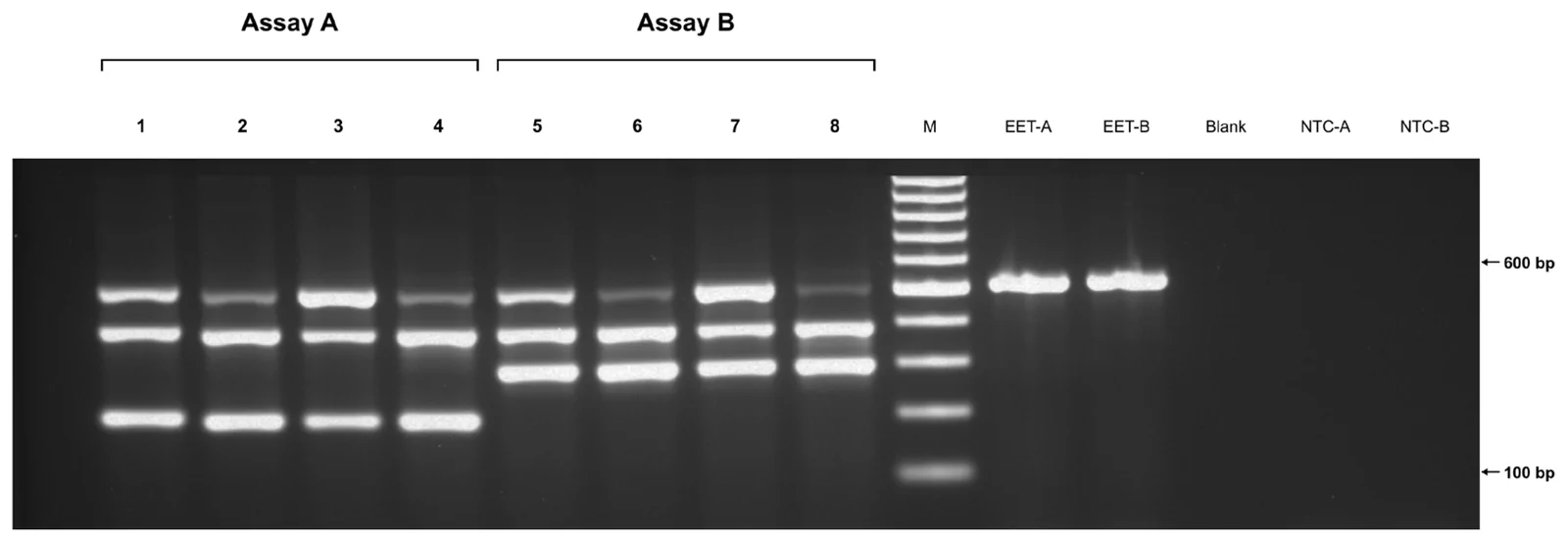
Representative multiplex RT-PCR profiles from the evaluation of two selected dual-target assays using four CaLRV-positive cacao accessions. The virus-specific amplicons represented RdRP (389 bp) and MP (201 bp) in Assay A, and RdRP (389 bp) and CP/readthrough (296 bp) in Assay B; the cacao *ACP1* internal-control amplicon (IC) was 508 bp. Within Assay A and Assay B, lanes 1–4 and 5–8, respectively, denote cacao accessions LCT EEN 412, TSH 1188, POUND 15/A, and TSAN 792 in the same order. EET-A and EET-B denote the CaLRV-negative cacao accession EET 272 [ECU] tested using Assays A and B, respectively; NTC-A and NTC-B, the corresponding no-template controls; Blank, unloaded lane; M, GeneRuler 100 bp DNA Ladder. RdRP, RNA-dependent RNA polymerase; MP, movement protein; CP, coat protein; *ACP1*, acyl carrier protein 1.

Both assays generated the two expected CaLRV-specific products together with the *ACP1* product in each of the four positive accessions. The CaLRV-negative cacao control generated only the *ACP1* product, and the NTCs remained negative. Assay A was retained as the primary screening configuration, whereas assay B was retained as a complementary configuration for investigating discordant or otherwise uncertain results (Figure 6).

### 3.9. No Evidence of CaLRV Transfer Under the Pruning Conditions Tested

Before exposure, all eight Amelonado seedlings tested negative for CaLRV, whereas the TSH 1188 source plant tested positive. All eight recipient seedlings subsequently remained CaLRV-negative at every three-month sampling interval through 24 months. The cacao IC was detected in every sample, confirming successful RNA extraction, reverse transcription, and amplification, and all assay controls produced the expected results (Supplementary Table S10). No evidence of CaLRV transfer was detected under the conditions tested.

## 4. Discussion

This study demonstrates why molecular diagnostics should be reassessed as pathogen diversity becomes better represented. Previous studies established the occurrence, genome organisation, and initial molecular detection of CaLRV [2,6,7]. The present study extends that foundation by identifying substantially greater genomic variation, including the divergent CaLRV-LCTEEN412 genome, and demonstrating its diagnostic consequences. The principal advance is the progression from expanded genomic representation and recognition of diagnostic limitations to assays designed around the currently known diversity.

The distribution of positive accessions provides information about germplasm movement pathways, although the screening results should not be interpreted as estimates of field prevalence because the collection was not assembled by random sampling. All five recently received pre-quarantine accessions from CATIE tested positive. This result is consistent with the earlier observation that CaLRV-positive accessions within a chronologically examined CATIE-derived panel had been received at ICQC-R after 2010 [2]. CaLRV sequences were also recovered previously from public RNA-seq datasets generated from cacao maintained at CATIE [6]. Together, these observations are consistent with CATIE-origin material representing one potential pathway by which the common CaLRV lineage may have moved between international germplasm collections. However, they do not establish the direction or timing of transmission, the point at which individual accessions became infected, or the origin of the lineage. Nor do they imply that all cacao germplasm maintained at CATIE is infected.

Detection of CaLRV in all five accessions while they were still in pre-quarantine also illustrates the operational value of molecular screening. The ICQC-R quarantine procedure [26] includes grafting onto indicator seedlings followed by weekly visual inspection over a two-year period, while current safe-movement guidance recommends PCR-based screening in combination with visual indexing [3]. Molecular testing can therefore identify CaLRV before completion of the prolonged graft-indexing period and inform early quarantine decisions. It should be regarded as a complement to, rather than a replacement for, the established visual and graft-based procedures.

The screening of older material provides an important contrast. Only one of 174 accessions attributed to Trinidad and Tobago was positive, and this was the recently received quarantine accession POUND 15/A. The absence of CaLRV from the other 173 accessions does not support widespread infection among the older Trinidad and Tobago-derived holdings. CaLRV was also detected in TSAN 792 and VB 681, both received from CEPLAC/CEPEC, Brazil, in 2000. Their shared donor and receipt year, the restricted occurrence of CaLRV among post-quarantine accessions, and the absence of detected known or presumptive vectors during routine monitoring support the interpretation that these infections pre-dated arrival at ICQC-R rather than arose within the collection. The available evidence does not determine whether the virus was endemic in Brazil, present at CEPLAC/CEPEC, or acquired earlier in the propagation and exchange history of these accessions.

CaLRV-LCTEEN412 represents a separate epidemiological and genomic observation. Its marked divergence was independently supported by metatranscriptomic sequencing and long-amplicon verification. Conservation of the expected genome organisation, translational features, and intact coding regions further argues against assembly or annotation error. LCT EEN 412 was received directly from Ecuador in 2005, and no closely related genome was identified among the complete or near-complete CaLRV sequences analysed. Regular monitoring of the quarantine collection did not detect any known or presumptive vector, and no transfer was detected in the pruning experiment. Together, these observations make acquisition of this lineage within the quarantine collection less likely and support the interpretation that it was already associated with LCT EEN 412 before importation. Until related sequences are detected through independent sampling, the lineage should be described as Ecuador-associated rather than assumed to be widespread or to have originated within Ecuador.

Reanalysis of the host-focused datasets in BioProject PRJNA558793 [27] yielded one near-complete genome and sequence information from four additional accessions, illustrating the value of repurposing public plant transcriptomes for virus discovery. Differences in assembly completeness could reflect library preparation, sequencing depth, tissue selection, virus titre, or assembly performance and should not be interpreted as biological genome deletions or direct measures of infection intensity. Including the fragmented sequences in primer-site assessment, while excluding them from whole-genome identity and variant-count analyses, allowed their informative regions to contribute to assay design without overstating assembly completeness.

Whole-genome comparisons showed that the previously available genomes and most genomes examined here belong to a closely related group, whereas CaLRV-LCTEEN412 shares only approximately 85% nucleotide identity with that group. Variation was distributed unevenly across the genome, and the relative conservation of individual features differed between nucleotide and amino acid comparisons. Protein conservation may indicate functional constraint, but it does not ensure conservation of the underlying nucleotide sequence required for primer annealing, because synonymous substitutions can preserve a protein sequence while altering primer-binding sites. The overlapping reading frames of ORF3 and ORF4 further complicate interpretation because a substitution can have different coding consequences in the two products. Primer selection must therefore be based on nucleotide alignments rather than inferred from protein conservation or biological function alone. This conclusion is consistent with broader analyses showing that sequence variation is not distributed uniformly across polerovirus genomes [4].

The current ICTV classification places cacao leafroll virus in the species *Polerovirus CALRV*, represented by OR423049 [5]. Relative to OR423049, CaLRV-LCTEEN412 exceeded 10% amino-acid divergence in P0, P1, the P1–P2 fusion protein, and the P3–P5 readthrough protein, satisfying one of the sequence-based species-demarcation indicators used for poleroviruses; however, its whole-genome nucleotide divergence was approximately 15%, below the approximately 25% criterion [5]. These indicators should not be interpreted in isolation. CaLRV-LCTEEN412 is represented by a single genome from one cacao accession, independently sampled relatives are unavailable, and comparative host-range, cross-protection, serological, and vector-specificity data have not been determined. We therefore retain it provisionally as a divergent CaLRV variant or lineage while recognising that its formal taxonomic placement should be reassessed when additional genomic and biological evidence becomes available.

The two GARD analyses indicated that a single phylogenetic history did not adequately describe the complete genome alignment. Excluding CaLRV-LCTEEN412 substantially reduced the number of candidate breakpoint positions considered and shifted their inferred positions, even though nine putative breakpoints were retained in both analyses. This sensitivity to dataset composition, together with the small number of available genomes and the high similarity among nine of them, means that the results should be interpreted as exploratory evidence of phylogenetic heterogeneity rather than confirmation of particular recombination events or parental lineages. GARD identifies changes in phylogenetic signal and does not by itself prove a particular historical recombination event [22]. Neither analysis placed a breakpoint within the coat protein region, providing a reasonable basis for analysing that region as a single phylogenetic partition; confirmation of genome-wide recombination patterns will require more sequences and complementary analytical methods.

The coat-protein analyses also clarify the appropriate use of this marker. The amino acid tree placed all CaLRV sequences in a strongly supported clade distinct from the related viruses, and both the amino acid and nucleotide analyses separated CaLRV-LCTEEN412 from the closely related CaLRV group. The coat protein is therefore suitable for confirming CaLRV affiliation and recognising the divergent lineage. However, extensive identity among the closely related sequences left most relationships within the common group unresolved. Coat-protein phylogeny should not therefore be used to infer detailed transmission relationships among these accessions. Whole-genome data, or more variable regions assessed for recombination, will be required for higher-resolution epidemiological analysis.

The performance of the previously published assays should be interpreted in relation to the sequence information available when they were developed. Those assays provided the first practical molecular tools for detecting CaLRV [2] and were not designed with knowledge of the LCT EEN 412 lineage. The expanded alignment revealed multiple mismatches in some published CaLRV-specific primer-binding sites, including mismatches close to the 3-prime ends of primers. The discordant amplification of LCT EEN 412 was consistent with these sequence differences. Mismatches were also identified in some primers previously used for broader polerovirus detection or CaLRV genome characterisation, indicating that the diagnostic implications extend beyond a single published assay. Comparable problems have been documented beyond cacao: conserved-site primers showed genotype-dependent amplification bias in citrus tristeza virus, and primer mismatches caused inconsistent RT-PCR detection of several berry-crop viruses until the assays were redesigned [28,29].

The effects of individual mismatches were not tested by evaluating every primer pair under matched experimental conditions. It would therefore be inappropriate to attribute assay failure conclusively to a particular substitution or to state that every mismatched primer will fail. Primer position, mismatch type, reaction conditions, and template concentration can all affect amplification [30,31]. The evidence supports the more precise conclusion that the newly identified variation creates a risk of inefficient amplification or false-negative detection, particularly where mismatches occur at or near the 3-prime terminus. This interpretation recognises a diagnostic limitation without suggesting that the earlier assays were incorrectly designed.

The expanded alignment was used prospectively to select conserved sites for the new assays. Conservation of these sites across the sequences included in the design alignment was therefore a design requirement rather than a subsequent discovery. Both assays combine a common RdRP target with a second viral target and a cacao internal control. The two-target format reduces dependence on a single viral locus, while the internal control distinguishes a valid negative result from failure of RNA extraction, reverse transcription, or amplification, an established advantage of multiplex plant-virus RT-PCR [32]. Importantly, the original three-assay panel did more than detect CaLRV: discordance among independent viral loci exposed LCT EEN 412 as an atypical variant that a single-locus result could have obscured. The new dual-target format retains this capability within one reaction: loss of one viral amplicon while the other and the IC remain positive should prompt repeat testing and sequence investigation, as target discordance has revealed divergent plant-pathogen variants in other systems [33]. Concordant absence of both CaLRV targets, however, cannot exclude a future variant carrying changes at both loci.

The assays serve complementary purposes: Assay A, targeting RdRP and MP, is suitable for primary screening, whereas Assay B, targeting RdRP and CP/readthrough, can be used to investigate discordant or otherwise uncertain results. Because both assays share the RdRP and internal-control components, assay B is not completely independent of assay A; its complementary value comes from the alternative downstream viral target. On the current evidence, routine application of both assays to every sample would add time and cost without a demonstrated diagnostic benefit when assay A produces a clear concordant result. A sequential strategy, with assay B reserved for discordant, uncertain, or high-consequence samples, is therefore the most proportionate use of the two formats.

The four-accession evaluation panel included LCT EEN 412 together with three accessions representing the closely related CaLRV group; geographic origin was not used as a proxy for viral diversity. Both assays detected all four positive accessions. These results demonstrate detection of the divergent CaLRV-LCTEEN412 lineage and the closely related CaLRV group represented in the current dataset, but do not establish universal detection of future variants or complete diagnostic performance. The assays should therefore be described as evaluated rather than comprehensively validated. Analytical sensitivity, specificity, and repeatability remain to be established, and the primer-binding sites should be reassessed as further CaLRV sequences become available.

The pruning experiment was included because routine pruning is unavoidable during long-term quarantine maintenance and procedure-specific evidence on incidental tool-mediated transfer is relevant to containment decisions. None of the exposed Amelonado seedlings became CaLRV-positive during 24 months of repeated testing, providing no evidence that CaLRV was transferred through the pruning procedure evaluated. This result is consistent with the phloem limitation of poleroviruses and the absence of established efficient transmission by routine mechanical inoculation [5,34]. Specialized procedures, including particle bombardment or inoculation with concentrated virions or viral RNA, can mechanically establish infection with some poleroviruses [35], but these procedures are not equivalent to incidental transfer on pruning tools. The experiment does not exclude every form of transmission because it involved one recipient genotype, eight plants, and one pruning procedure. It also did not assess graft, seed, root-contact, or vector transmission, all of which remain unresolved for CaLRV [3]. The conclusion should therefore remain restricted to the absence of detectable transfer under the specific conditions tested.

Regular visual monitoring remains an important component of quarantine inspection, but it cannot alone determine CaLRV infection status reliably. Previous studies have reported CaLRV in plants with and without visible abnormalities, and similar symptoms have also occurred in plants that tested negative [2,3]. No leaf rolling was observed in the CaLRV-positive accessions examined during this study; however, symptoms were not scored systematically. The present results therefore neither establish nor exclude a causal relationship between CaLRV and leaf rolling. Controlled studies are needed to determine whether symptom expression depends on viral lineage, cacao genotype, environmental conditions, coinfection, or other biotic or abiotic factors. The absence of a dependable symptom association supports combining regular visual inspection with molecular testing during germplasm movement and quarantine assessment.

Several limitations define the boundaries of the study. The germplasm panel was non-random, the complete-genome comparison contained only ten sequences, and CaLRV-LCTEEN412 was the sole representative of the divergent phylogenetic group. Four public assemblies were fragmented, and the recombination evidence remains preliminary. Assay evaluation was limited to four positive accessions and did not establish analytical sensitivity or repeatability. The study also did not establish field prevalence, pathogenicity, vector identity, or graft and seed transmissibility. These limitations do not undermine the diagnostic advance, but they prevent claims of universal assay coverage or definitive geographic and evolutionary reconstruction.

Future work should prioritise sequencing of CaLRV from independently sampled cacao in Ecuador, Costa Rica, Brazil, and Trinidad and Tobago. Ecuadorian sampling is particularly important for determining whether LCT EEN 412 represents an isolated variant, a broader regional lineage, or a taxonomically distinct population. Diagnostic development should include analytical limits of detection, repeatability, testing against a larger blinded panel, and continued in silico assessment as new genomes become available [36]. The broader lesson is that sequence-based assays should be treated as evolving tools. Their performance should be reassessed periodically as the genomic representation of the target virus expands.

In conclusion, the expanded genomic dataset revealed a level of CaLRV diversity that was not represented when the original assays were developed. The divergent CaLRV-LCTEEN412 lineage exposed the diagnostic consequences of this incomplete representation and provided the rationale for a dual-target approach. The resulting assays detected all four positive accessions evaluated and provide both an internal control for extraction, reverse transcription, and amplification and an alternative downstream target for complementary testing. The study therefore demonstrates that the practical value of virus sequencing lies not only in documenting diversity, but in using that diversity to make better diagnostic decisions for the safe movement and conservation of cacao germplasm.

## Figures and Tables

**Table 1:** Summary of the primers used in cacao leafroll virus dual-target multiplex RT-PCR assays.

Set	Primer	Sequence (5′ to 3′)	OR423049 Coordinates	Target	Product (bp)
1	CaPV_2938_F2	ATAGTGCCCACCGACTGTTC	2949–2968	RdRP	389
	CaPV_3326_R2	GAACAGAATTCCAGATCTCGTG	3316–3337		
2	PolevirusMP_F	AGGGAGCAACAGCTTCACAT	3966–3985	MP	201
	PolevirusMP_R	GAGATGGAGCCTGATGTCGT	4147–4166		
3	CaPV_4260_F3	ACGGGTTAGAATGGCTCGAT	4271–4290	CP/readthrough	296
	CaPV_4555_R3	GGAGTCCCACTCGTTGTCAG	4547–4566		
4	ACP1_43_F1	GCACTCCGTCGTTTTTCATT	N/A	Cacao *ACP1*	508
	ACP1_550_R1	ACATGGGCAAATGAATCTCC	N/A		

**Table 2:** Cacao accessions that tested positive for cacao leafroll virus.

Accession Name	RUQ Accession	Donor Collection	Quarantine Status	Year Received
TSAN 792	RUQ 1031	CEPLAC/CEPEC (Brazil)	Post-quarantine	2000
VB 681	RUQ 1037	CEPLAC/CEPEC (Brazil)	Post-quarantine	2000
LCT EEN 412	RUQ 1554	EETP (Ecuador)	Post-quarantine	2005
POUND 15/A	RUQ 1765	ICG,T (Trinidad and Tobago)	Quarantine	2022
ICS 95	RUQ 1773	CATIE (Costa Rica)	Pre-quarantine	2025
PCMT 58	RUQ 1774	CATIE (Costa Rica)	Pre-quarantine	2025
CATIE-R 4	RUQ 1776	CATIE (Costa Rica)	Pre-quarantine	2025
CATIE-R 6	RUQ 1777	CATIE (Costa Rica)	Pre-quarantine	2025
CATIE-R 91	RUQ 1783	CATIE (Costa Rica)	Pre-quarantine	2025

Note: RUQ, University of Reading quarantine accession; CATIE, Tropical Agricultural Research and Higher Education Center; CEPLAC/CEPEC, Executive Commission of the Cocoa Farming Plan/Cocoa Research Center; EETP, Estacion Experimental Tropical Pichilingue; ICG,T, International Cocoa Genebank, Trinidad.

**Table 3:** Genomic feature lengths and pairwise identities of CaLRV genomes relative to OR423049.

Feature	Product or Region	Metric	OR423049	PZ760203 (TSAN 792)	PZ760204 (POUND 15/A)	PZ760205 (LCT EEN 412)	CaLRV-PA16
5′ UTR	Noncoding	Length (nt)	101	96	101	103	90
		Identity (nt, %)	100	97.92	90.10	52.43	98.89
ORF0	P0, silencing suppressor	Length (nt/aa)	690/229	690/229	690/229	687/228	690/229
		Identity (nt/aa, %)	100/100	98.41/97.38	97.54/96.51	82.75/78.17	98.26/97.38
ORF1	P1	Length (nt/aa)	2061/686	2061/686	2061/686	2079/692	2061/686
		Identity (nt/aa, %)	100/100	98.20/98.10	98.06/97.67	79.84/77.70	98.84/98.54
ORF1-ORF2	P1-P2, RNA-dependent RNA polymerase	Length (nt/aa)	3339/1112	3339/1112	3339/1112	3357/1118	3339/1112
		Identity (nt/aa, %)	100/100	98.65/98.38	98.71/98.20	83.87/82.16	99.19/99.01
Intergenic region	Noncoding region between ORF2 and ORF3a	Length (nt)	94	94	94	97	94
		Identity (nt, %)	100	98.94	98.94	76.53	98.94
ORF3a	P3a, putative movement protein	Length (nt/aa)	123/40	123/40	123/40	123/40	123/40
		Identity (nt/aa, %)	100/100	99.19/100	99.19/97.50	90.24/92.50	100/100
ORF3	P3, coat protein	Length (nt/aa)	606/201	606/201	606/201	603/200	606/201
		Identity (nt/aa, %)	100/100	99.67/99.00	100/100	92.61/93.07	100/100
ORF4	P4, movement protein	Length (nt/aa)	405/134	405/134	405/134	411/136	405/134
		Identity (nt/aa, %)	100/100	99.51/99.25	100/100	94.40/91.18	100/100
ORF3-ORF5	P3-P5, readthrough protein	Length (nt/aa)	2028/675	2028/675	2028/675	2010/669	2028/675
		Identity (nt/aa, %)	100/100	99.46/98.96	99.75/99.56	82.57/84.22	99.90/99.70
3′ UTR	Noncoding	Length (nt)	180	177	168	169	125
		Identity (nt, %)	100	100	98.81	85.96	100

Note: Coding-region nucleotide lengths include termination codons, whereas amino acid lengths exclude them. Pairwise nucleotide and amino acid identities were calculated relative to OR423049. Terminal UTR gaps were treated as missing data in identity calculations. CaLRV-PA16 denotes the near-complete assembly recovered from the public PA 16 dataset. CaLRV, cacao leafroll virus; nt, nucleotides; aa, amino acids; UTR, untranslated region; ORF, open reading frame.

## Data Availability

The metatranscriptomic raw reads and genome assemblies generated in this study are available in NCBI under BioProject PRJNA1503048: TSAN 792 (GenBank PZ760203; BioSample SAMN62002539; SRA SRR39866085), POUND 15/A (PZ760204; SAMN62002540; SRR39866084), and LCT EEN 412 (PZ760205; SAMN62002541; SRR39866083). Public cacao RNA-seq reads analysed here are available under BioProject PRJNA558793 (SRR9938245, SRR9938323, SRR9938250, SRR9938307, and SRR9938227). The derived PA 16 near-complete assembly and ten CaLRV contigs recovered from four additional datasets are provided in Supplementary Data S1. The plant-virus-focused protein database used for viral-contig identification is provided in Supplementary Data S2; supporting comparison data are provided in Supplementary Tables S1–S12. The nanopore insert consensus used for independent verification of CaLRV-LCTEEN412 is provided in Supplementary Data S3; raw nanopore reads were not available to the authors.

## Supplementary Materials

The following supporting information can be downloaded at https://www.mdpi.com/article/10.3390/pathogens15090975/s1, Supplementary Method S1: Terminal assessment and independent sequence verification of the CaLRV-LCTEEN412 assembly; Supplementary Figure S1: RT-PCR assessment of the near-terminal regions of the CaLRV-LCTEEN412 assembly using three cDNA preparations; Supplementary Tables S1–S12: Complete germplasm screening data, public-data assemblies [17], identity and variant analyses, recombination results, primer-site analyses [2,6,7,24], assay optimisation, mechanical-transfer testing, and primer sequences; Supplementary Data S1: CaLRV assemblies recovered from public cacao RNA-seq data; Supplementary Data S2: Plant-virus-focused protein database used for DIAMOND BLASTX searches. Supplementary Data S3: CaLRV-LCTEEN412 nanopore insert consensus.
