# Supplementary material for: Expanded Genomic Diversity of Cacao Leafroll Virus Enables Improved Detection Using Multiplex RT-PCR
Source: Pathogens. 2026 Sep 14;15(9):975. doi: 10.3390/pathogens15090975 (PMC13610897; doi:10.3390/pathogens15090975)
Supplement: Supplementary file 1 [file pathogens-15-00975-s001.zip › Supplementary_Method_S1_and_Figure_S1.pdf]

Supplementary Method S1. Terminal assessment and independent sequence verification of the CaLRV-LCTEEN412 assembly

Seven overlapping primer pairs were designed from the CaLRV-LCTEEN412 assembly to assess the near-terminal regions. Three pairs covered positions 38–306 near the 5′ end, and four pairs covered positions 5665–5983 near the 3′ end. Predicted products ranged from 145 to 213 bp. Primer sequences, coordinates, and expected product sizes are provided in Supplementary Table S12.

The primer pairs were evaluated using three LCT EEN 412 cDNA preparations. Routine cDNA was synthesised using SuperScript IV VILO Master Mix with ezDNase Enzyme, as described in Section 2.1 of the main manuscript. Two additional gene-specific cDNAs were synthesised from 1 μg of total RNA using the RevertAid First Strand cDNA Synthesis Kit and either L412_5926R5 or L412_5983R6 as the reverse-transcription primer. Each 20 μL reaction contained 10 pmol of the selected primer, 1 μL of 10 mM dNTP mix, 4 μL of 5× reaction buffer, 1 μL of RevertAid H Minus reverse transcriptase, total RNA, and nuclease-free water to volume. Reverse transcription was conducted at 62.5 °C for 40 min, followed by enzyme inactivation at 70 °C for 10 min. The resulting cDNA was diluted 1:4 with nuclease-free water.

The L412_5926R5-derived cDNA was selected for near-complete-genome amplification. The L412_5983R6-derived cDNA was included to assess the most distal primer pair spanning positions 5839–5983, whose reverse-primer site lies downstream of L412_5926R5.

Each 20 μL terminal-region PCR contained 10 μL of Platinum II Hot Start PCR Master Mix, 1.5 μL of the selected terminal primer-pair mixture (each primer at 5 μM), 5 μL of cDNA, and 3.5 μL of nuclease-free water. Thermal cycling comprised initial denaturation at 94 °C for 2 min; 35 cycles of 94 °C for 10 s, 60 °C for 10 s, and 68 °C for 20 s; and final extension at 68 °C for 2 min. Products were resolved by agarose-gel electrophoresis and compared with a GeneRuler 100 bp DNA Ladder (Thermo Fisher Scientific).

The previously reported movement-protein/ACP1 multiplex assay was included as a CaLRV amplification control. ACP1 amplification was expected from VILO cDNA but not from cDNA synthesised using a virus-specific reverse-transcription primer. A separate no-template control was included with each cDNA-preparation series. The gel layout and lane assignments are provided in Supplementary Figure S1.

For near-complete-genome amplification, cDNA synthesised using L412_5926R5 as the reverse-transcription primer was amplified with the L412_38F1/L412_5926R5 primer pair. This primer pair was designed to amplify a 5889-nt region spanning positions 38–5926 of the CaLRV-LCTEEN412 assembly. Each 25 μL reaction contained 12.5 μL of Phusion Flash PCR Master Mix, 2 μL each of 5 μM L412_38F1 and L412_5926R5, 5 μL of gene-specific cDNA, and 3.5 μL of nuclease-free water. Thermal cycling comprised initial denaturation at 98 °C for 10 s; 35 cycles of 98 °C for 1 s, 60 °C for 5 s, and 72 °C for 150 s; and final extension at 72 °C for 3 min. The product was resolved on a 1% agarose gel, stained with ethidium bromide, and compared with a GeneRuler 1 kb DNA Ladder (Thermo Fisher Scientific).

The amplicon of the expected size was excised and purified using the GeneJET Gel Extraction Kit (Thermo Fisher Scientific, UK) and cloned using the Zero Blunt TOPO PCR Cloning Kit for Sequencing (Thermo Fisher Scientific, UK). Recombinant plasmid DNA was subjected to nanopore long-read sequencing by Source BioScience (Nottingham, UK). The resulting insert consensus sequence was aligned with the metatranscriptomic CaLRV-LCTEEN412 assembly to determine sequence coverage and nucleotide identity.

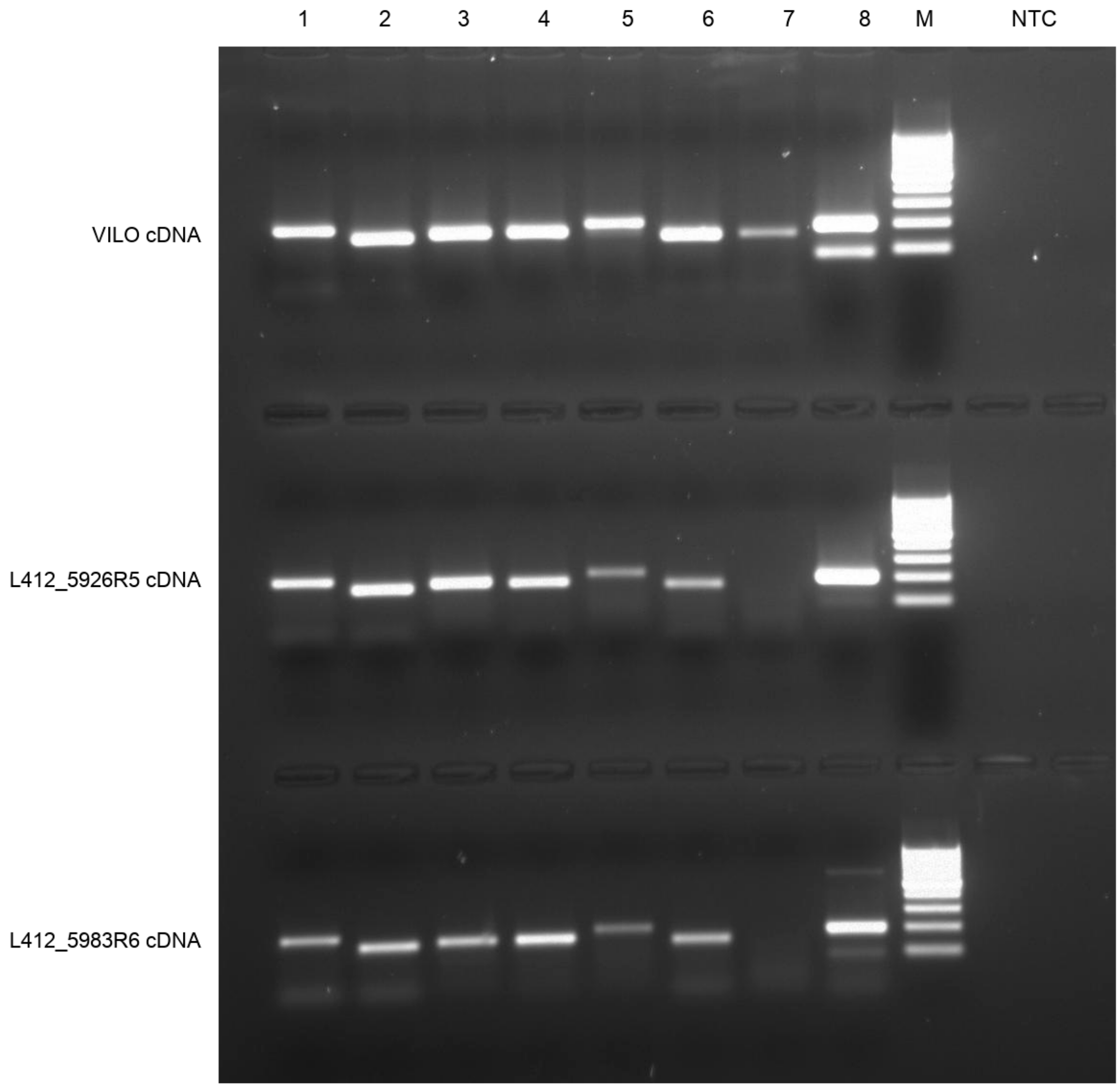

Supplementary Figure S1. RT-PCR assessment of the near-terminal regions of the CaLRV-LCTEEN412 assembly using three cDNA preparations. The upper, middle, and lower rows show VILO cDNA, L412_5926R5-derived cDNA, and L412_5983R6-derived cDNA, respectively. Within each row, lanes 1–7 correspond to terminal primer pairs 1–7 listed in Supplementary Table S12, and lane 8 contains the previously reported movement-protein/*ACP1* multiplex control. M, GeneRuler 100 bp DNA Ladder; NTC, no-template control. In the middle row, lane 7 was not assessable because the L412_5926R5-derived cDNA did not extend to the downstream L412_5983R6 binding site.
